# Cerebrospinal Fluid from Restless Legs Syndrome Patients Reduces Iron Uptake in Blood–Brain Barrier Endothelial Cells by Disrupting the Regulation of Transferrin Receptors

**DOI:** 10.1002/ana.78221

**Published:** 2026-04-07

**Authors:** Kondaiah Palsa, Aurosman Pappus Sahu, David B. Rye, Lynn Marie Trotti, Irina A. Elcheva, Vladimir S. Spiegelman, James R. Connor

**Affiliations:** ^1^ Department of Neurosurgery Penn State College of Medicine Hershey PA USA; ^2^ Department of Neurology and Emory Sleep Center Emory University School of Medicine Atlanta GA USA; ^3^ Division of Pediatric Hematology and Oncology, Department of Pediatrics Penn State College of Medicine Hershey PA USA

## Abstract

**Objective:**

Restless legs syndrome (RLS) is a sensorimotor disorder marked by an uncontrollable urge to move the legs. A pathophysiological hallmark of RLS is brain iron deficiency. The endothelial cells (ECs) of the blood–brain barrier (BBB) are responsible for regulating brain iron uptake. Our objective is to determine if brain iron uptake is altered in ECs in RLS.

**Methods:**

Human ECs were generated from induced pluripotent stem cells (iPSCs). ECs were exposed to RLS (n = 14) or control cerebrospinal fluid (CSF) (n = 15), and ^57^Fe‐Transferrin transport was determined. Immunoblotting and quantitative polymerase chain reaction were used to analyze protein, microRNA (miRNA), and messenger RNA (mRNA) expressions. We performed miRNA and transferrin receptor 1 (TfR1) 3′ iron‐responsive elements (IRE) interaction in HEK‐293 cells by using a pMIR‐REPORTER luciferase vector.

**Results:**

Free and protein‐bound iron in CSF from RLS subjects were decreased compared to controls. Exposure of ECs to RLS CSF significantly decreased the uptake and transport of ^57^Fe compared to the control. TfR1 expression decreased while iron regulatory proteins (IRP) increased in RLS‐CSF exposed ECs. TfR1 expression was also regulated by miRNAs. The miR‐124‐3p levels were higher in RLS CSF‐derived extracellular vesicles than in the control. It binds the TfR1 3′ IRE sequence in the pMIR‐REPORTER vector, reducing luciferase expression.

**Interpretation:**

When ECs are treated with CSF from RLS patients, they show a profile of iron deficiency, except that the TfR1 expression does not increase as would be predicted. The decrease in TfR1 protein expression is because of a reduction in TfR1 mRNA stability by the binding of increased miR‐124‐3p. ANN NEUROL 2026;100:48–58

## Graphical abstract

Model figure of BBBECs TfR1 regulation in control and RLS: Graphical representation of TfR1 regulation in ECs by IRPs and representing it can be dysregulated by miR‐124‐3p in ECs of RLS. FPN1, ferroportin; BBBEC, blood‐brain barrier endothelial cells; IRP, iron regulatory proytein1/2; TfR1, Transferrin receptor; IRE, Iron responsive elements; Tf, transferrin; EVs, extracellular vesicles.

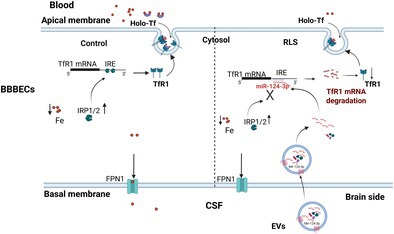

Restless legs syndrome (RLS) is a sensorimotor disorder affecting approximately 5% to 10% in the general population.^1^, ^2^, ^3^ The principal feature of RLS is an urge to move the legs in association with uncomfortable, even painful, leg sensations. The symptoms intensify on sitting or lying, which often prompts movement or walking, which brings various degrees of symptomatic relief.^4^ Brain iron insufficiency is reported as part of the pathophysiology of RLS.^5^, ^6^, ^7^, ^8^ Peripheral iron deficiency (ID) is reported to increase the prevalence of RLS.^9^ However, even in RLS patients with normal peripheral iron stores, brain autopsy analyses, magnetic resonance imaging‐based brain iron determination, and cerebrospinal fluid (CSF) analysis have all demonstrated the presence of brain iron deficiency.^10^ Therefore, brain iron deficiency is considered one of the primary pathologies in RLS. The brain iron levels are regulated by the blood–brain barrier endothelial cells (BBBECs)^11^ but the regulation or possible dysregulation of iron uptake at blood–brain barrier (BBB) in RLS remains unclear.

Our previous in vitro studies demonstrated that BBBECs are critical regulators of iron transport to the brain.^12^, ^13^ At BBB, transferrin‐bound iron binds to the transferrin receptor1 (TfR1) on the luminal surface of the endothelial cells (ECs) and is subsequently endocytosed. The endocytosed iron is transported either as free iron via ferroportin1/hephaestin (FPN1/HEPH), an iron exporter, or as a Tf‐iron complex into the brain parenchyma.^12^, ^14^, ^15^ We recently discovered that exosomes are involved in iron transport from ECs, and the release of exosomes is regulated by the ECs iron concentration.^16^ The labile iron pool within the ECs is regulated by iron regulatory proteins (IRPs), which bind to iron‐responsive element (IRE) sequences in the 3′ messenger ribonucleic acid (mRNA) of TfR1 or 5′ of ferritin.^17^ The TfR1 mRNA, which has 3′ IRE sequences, is stabilized by the IRPs, whereas with ferritin, the 5′ IRE sequence IRP binding inhibits the translation of mRNA.^18^, ^19^, ^20^ Furthermore, TfR1 can also be post‐transcriptionally regulated by microRNAs (miRNAs), independent of cellular iron concentrations.^21^, ^22^, ^23^

We have published the paradigm‐shifting concept that the brain regulates brain iron uptake.^14^ Although BBBECs were previously thought to serve as passive conduits for iron, allowing it to flow through to the brain without regulation, our laboratory has shown that the uptake and release of iron are regulated at the BBB.^12^ It follows that signals from the extracellular fluid in the brain (including CSF) direct the iron transporters in ECs involved in iron transport across the BBB.^14^ However, in RLS CSF iron levels are reportedly lower than normal.^8^ We hypothesize that some signals in CSF must dysregulate the iron uptake or release in the BBBECs in RLS patients. Therefore, the present set of studies uses a human EC culture model along with control and RLS patient CSF to determine whether RLS CSF decreases the iron transport to the brain by dysregulating iron uptake in BBBECs.

## Subjects and Methods

### RLS CSF Collection and Patient Details

CSF samples were obtained from a parent study evaluating patients with excessive daytime sleepiness. CSF was collected via lumbar puncture at L3–L4 or L4–L5 during daytime hours (8 am to 5 pm) and then aliquoted into 1.0ml tubes for storage at −80°C.

For inclusion in the parent study, participants providing CSF had to have a diagnosis of narcolepsy, idiopathic hypersomnia, or subjective sleepiness (without objective testing abnormalities). For inclusion in the present study, participants providing CSF had to be: (1) women, and (2) evaluated for the presence or absence of RLS through face‐to‐face evaluation by a board‐certified sleep medicine physician with expertise in the assessment of RLS and following diagnostic criteria from the International Classification of Sleep Disorders and International Restless Legs Syndrome Study Group. Exclusion criteria for both studies were contraindication for lumbar puncture or inability/unwillingness to provide consent. All participants provided written informed consent before participating in the parent study in line with the Declaration of Helsinki, the parent study and this sub‐study were approved by the Emory University institutional review board.

This study included 14 women with RLS and 15 women without RLS, who served as controls. The mean ± standard deviation (SD) age of participants was 32.7 ± 11.7 years, without significant differences between groups (RLS group 34.9 ± 14.1 vs controls 30.7 ± 8.9, *p* = 0.33). The hematological parameters of hemoglobin, serum iron, ferritin, and transferrin saturation were similar in the RLS participants and controls (Table 1). Two RLS participants and 2 controls were taking an oral iron supplement at the time of their lumbar puncture. Five of 14 RLS participants were on RLS medications at the time of their lumbar puncture (1 dopamine agonist, 1 levodopa, 2 α‐2‐Δ ligands, and 1 opioid).

**TABLE 1:** Control and RLS Patient's Hematological Parameters

Characteristic	Controls, n = 15	RLS, n = 15	*p*
Age, yr	30.7 ± 8.9	34.9 ± 14.1	ns
Hemoglobin, g/dL	13.2 (3.9)	13.3 (0.9)	ns
Serum ferritin, ng/mL	30.3 (21.0)	62.5 (87.7)	ns
Serum iron, μg/dL	85.2 (18.3)	79.8 (34.3)	ns
Transferrin saturation, %	21.4 (5.1)	23.0 (9.7)	ns

RLS = restless legs syndrome. ns = non significant.

### Human Induced Pluripotent Stem Cells‐Derived Human BBBECs

Human induced pluripotent stem cells (hiPSCs) (ATCC‐DYS0100, ATCC, (American Type Culture Collection), United States of America (USA)) were differentiated into human BBBECs as stated before.^16^, ^24^ Briefly, 6‐well plates were coated with Matrigel and incubated for at least 4 hours in a 5% CO₂ incubator. hiPSCs were then seeded at a density of 18,000 cells/cm^2^ in E8 medium supplemented with 10nM ROCK inhibitor (Y‐27632, R&D system, USA) onto the matrigel‐coated plates. After 24 hours, the medium was replaced with E6 medium (Thermo Fisher Scientific, USA, A1516401), which was changed every 24 hours for 4 days. On day 4, cells were cultured in endothelial serum‐free medium (Thermo Fisher Scientific, 11111) supplemented with 10nM basic fibroblast growth factor (bFGF) (Peprotech, USA,100–18B), 10μM retinoic acid (Sigma, USA, R2625), and 1% B27 (Thermo Fisher Scientific, USA, 17504–044) for 48 hours. Following differentiation, ECs were subcultured onto collagen IV and fibronectin‐coated inserts in the same medium. To promote barrier formation and enhance transendothelial electrical resistance (TEER), the medium was replaced after 24 hours with endothelial serum‐free medium containing 1% B27.

### ^57^Fe‐Tf Transport

Preparation and transport of ^57^Fe‐Tf were conducted as per the prior description.^16^, ^25^ Briefly, 0.4μm Transwell inserts were coated with a mixture of water, collagen IV (1mg/mL), and fibronectin (1mg/mL) at a 5:4:1 ratio and incubated for at least 4 hours in a 5% CO₂ incubator. After removal of the coating solution, differentiated ECs were subcultured onto the coated inserts in endothelial serum‐free medium supplemented with 1% B27, bFGF, and retinoic acid on the apical side, with 1.5mL of medium added to the basal chamber. After 24 hours, the medium was replaced with endothelial serum‐free medium containing only 1% B27 and incubated for an additional 24 hours. Before the ^57^Fe–Tf transport assay, cells were washed with phosphate buffered saline (PBS), and endothelial serum‐free medium was added to both chambers (0.5mL apical, 1.5mL basal). TEER was measured using a volt/ohm meter to assess barrier integrity and tight junction permeability; TEER values over all experiments averaged 3,800 ± 126 Ω × cm^2^. During transport experiments, rhodamine B isothiocyanate‐dextran (70kDa; Sigma) was added to the apical chamber to assess ECs permeability.

Control and RLS CSF were diluted with human ECs serum‐free medium to a concentration of 25%. The CSF samples were added to the basal chamber and Tf ‐^57^Fe (1mg/mL) complex to the apical chamber, and cells were incubated at 37°C, 5% CO_2_ for 24 hours. Finally, ^57^Fe concentration was measured at 24 hours. Additionally, cell lysates were collected to measure protein and mRNA expression in respective conditions. For cell culture exposure studies, we pooled 2 samples of CSF and exposed them to the ECs. We pooled the n = 2 CSF samples in control or RLS based on their iron concentrations (for all pooled conditions). We exposed ECs with lower, higher, or mean values of iron concentrations, n = 2 from each condition for a total of 3 different groups from control and RLS. Each dot in the figure represents n = 2 of control and RLS.

### ^56^Fe and ^57^Fe Analysis

Free and protein‐bound iron (^56^Fe) in the 800μL of CSF were separated by Sephadex‐G50 Quick Spin columns. The cell culture media and CSF (free and protein‐bound fractions) acid digestion were performed as described previously.^24^, ^26^ Briefly, the samples were digested overnight with nitric acid (0.2mL) at 60°C. Iron concentrations were quantified by inductively coupled plasma mass spectrometry (ICP‐MS) using an appropriate standards. Results are reported in ng/mL.

### Luciferase Assay

Luciferase assay was conducted as described in earlier studies.^21^, ^27^ All luciferase experiments were performed in HEK‐293 cells. First, we co‐transfected with 1μg pMIR‐REPORTER luciferase containing 700bp of TfR1 3′ IRE, 20nM of has‐mir‐124‐3p (miRNA mimic, Dharmacon, USA, C‐300592‐05‐0005), miRIDIAN, a negative control Dharmacon, (CN‐0010000‐01‐05), and 200ng of control vector of pRL Renilla Luciferase (Promega, USA, E2231). A dual luciferase assay (Promega) was performed 48 hours after co‐transfection. For CSF luciferase experiments, we co‐transfected the Renilla and luciferase vectors with control and RLS CSF in human embryonic kidney‐293 (HEK‐293) cells (total n = 12 from control and RLS, n = 2 pooled samples from control and RLS CSF; n = 6). For miRNA mimic experiments, we performed 3 independent experiments in triplicate and generated 9 luciferase experiments. In a dual luciferase assay, the Renilla luciferase was used to normalize the firefly luciferase. After normalization with Renilla luciferase, the RLS and miR‐124‐3p luciferase activity was normalized with their control luciferase activity.

### Quantitative polymerase chain reaction

After 24 hours of control and RLS CSF treatment to the basal side of ECs, total RNA was isolated by using TRIzol. Complementary DNA (cDNA) was subsequently synthesized from the isolated RNA, and quantitative polymerase chain reaction (qPCR) was performed by using the TfR1 (Thermo Fisher Scientific, ID‐Hs00951083_m1), cluster of differentiation 63 (CD63) (Thermo Fisher Scientific, ID‐Hs01041238_g1), and β‐actin (Thermo Fisher Scientific, ID‐Hs01060665_g1) probes according manufacturer's protocol. The β‐actin was used to normalize the target genes by the ΔCt method. The results were normalized to the control group for each experiment and expressed as mean ± SD. To perform the qPCR for miRNAs, we isolated the extracellular vesicles (EVs) from the 2mL/subject of CSF by using the commercial EV isolation kit (QIAGEN, USA, 76743) the manufacturer's protocol. Briefly, to remove the debris in the CSF, it was centrifuged for 10 minutes at 2,000*g* at 4°C. Next, 800μL of EVs isolation reagent was added to the supernatant of CSF and incubated overnight. Following incubation EVs were pelleted by centrifuging at 10,000*g* for 1 hour at 4°C. After isolation, the EVs concentration and their structure were measured by nanoparticle tracking analysis (NanoSight NS300) and transmission electron microscopy (JEOL‐1400, Japan), respectively.^16^ Subsequently, an RNA isolation kit (Norgen Biotek, Canda, 58000) was used to isolate the total RNA, including miRNA, from the CSF‐derived EVs. The TaqMan Advanced miRNA cDNA synthesis kit (Applied Biosystem, USA) was used to synthesize cDNA from the EVs isolated miRNA. Next, miR‐124‐3p advanced miRNA assay probes (Thermo Fischer Scientific, A25576) were used to perform qPCR of miR‐124‐3p miRNA expression.

### Immunoblotting

An immunoblot was performed as stated earlier.^16^ Briefly, the control and RLS CSF were exposed to the basal side of ECs and incubated for 24 hours. Following incubation, apical side ECs cells were washed with PBS, and cells were lysed by using radioimmunoprecipitation assay (RIPA) buffer containing protease inhibitor cocktail (Sigma). After that bicinchoninic assay was performed to measure the protein concentration, and sodium dodecyl sulfate‐polyacrylamide gel electrophoresis was performed by 4% to 20% Criterion tris‐glycine extended (TGX) gels loaded with 25μg of protein. Subsequently, transferred the gel‐separated proteins onto the polyvinylidene fluoride (PVDF) membrane and blocked with 5% milk powder in 0.1% tris‐buffered saline with Tween‐20 (TBST) buffer. The blocked PVDF membrane probed with TfR1 (Santa Cruz, USA, sc‐65882, 1:250), FPN1 (Alpha Diagnostic International, USA, MTP‐11S, 1:1,000), ferritin heavy chain (FTH1) (Cell Signaling Technology, USA, 4393S, 1:1,000), ferritin light chain (FTL) (Abcam, USA, ab69090; 1:1,000), CD63 (Thermo Fisher Scientific, 1:1,000), CD81 (cell signaling technology, 1:1000, 52892) IRP1and 2 (Cell Signaling Technology, 20272, 37135; 1:1,000), and β‐actin (Sigma A544, 1:1,000), incubated overnight at 4°C. The probed membranes were washed with 0.1% TBST, and horseradish peroxidase ‐conjugated mouse or rabbit secondary antibodies were added and incubated for 1 hour at room temperature. The enhanced chemiluminescence (Santa Cruz) reagent was used to see the bands by using Amersham Imager 600 (GE Amersham, United Kingdom).

### Statistical Analysis

The GraphPad Prism 9.0 software was used to analyze the statistics in this study. We used the student unpaired *t* test for comparing the 2 groups, free and protein‐bound iron, and RLS and control CSF‐exposed ECs experiments. We use 2‐way analysis of variance followed by Tukey's post hoc for the free and protein‐bound iron differences between the control and RLS CSF. The statistically significant result was considered *p* < 0.05. The results were expressed as mean ± SD.

## Results

### RLS CSF Has Lower Free and Protein‐Bound Iron

**FIGURE 1:**
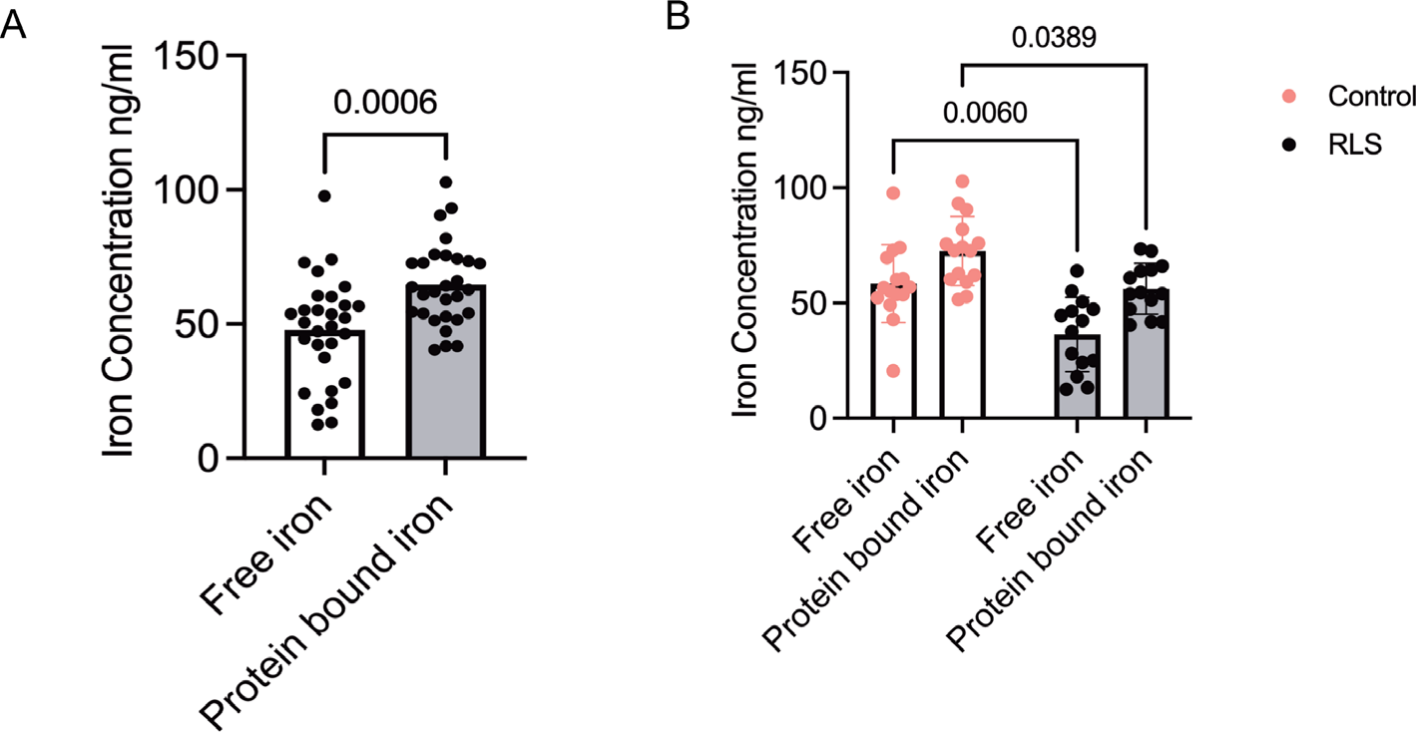
Restless legs syndrome (RLS) cerebrospinal fluid (CSF) has lower free and protein‐bound iron: the free and protein‐bound iron of CSF (800μL) was separated by the G‐50 Sephadox columns. After separation, we measured the free and protein‐bound iron by inductively coupled plasma mass spectrometry (ICP‐MS). (A) When all samples of CSF were combined, there is higher protein‐bound iron than free iron. n = 30, the data shown are the mean ± standard deviation (SD); unpaired *t* test. (B) RLS CSF has lower free and protein‐bound iron levels than controls. n = 14 for RLS, n = 15 for controls, the data shown are the mean ± SD, unpaired *t* test, and 2‐way analysis of variance with Tukey's post hoc test. [Color figure can be viewed at www.annalsofneurology.org]

Our in vitro studies showed that BBBECs release free and protein‐bound iron to the brain,^12^, ^16^ but levels of free and protein‐bound iron in human CSF have not been determined. To determine this, first, we separated the free and protein‐bound forms of iron by G‐50 columns, and iron concentration was measured by the ICP‐MS. We first examined the CSF from all patients and determined that free iron levels were significantly lower than the protein‐bound iron levels (Fig 1A). Previous studies showed the RLS CSF has lower iron levels than the controls,^5^, ^8^ however, the free versus protein‐bound iron levels in RLS CSF were not determined, so we examined that here. In RLS CSF, free and protein‐bound iron levels were significantly decreased compared to the controls (see Fig 1B).

### RLS CSF Reduces Iron Absorption and TfR1 Expression in the ECs

**FIGURE 2:**
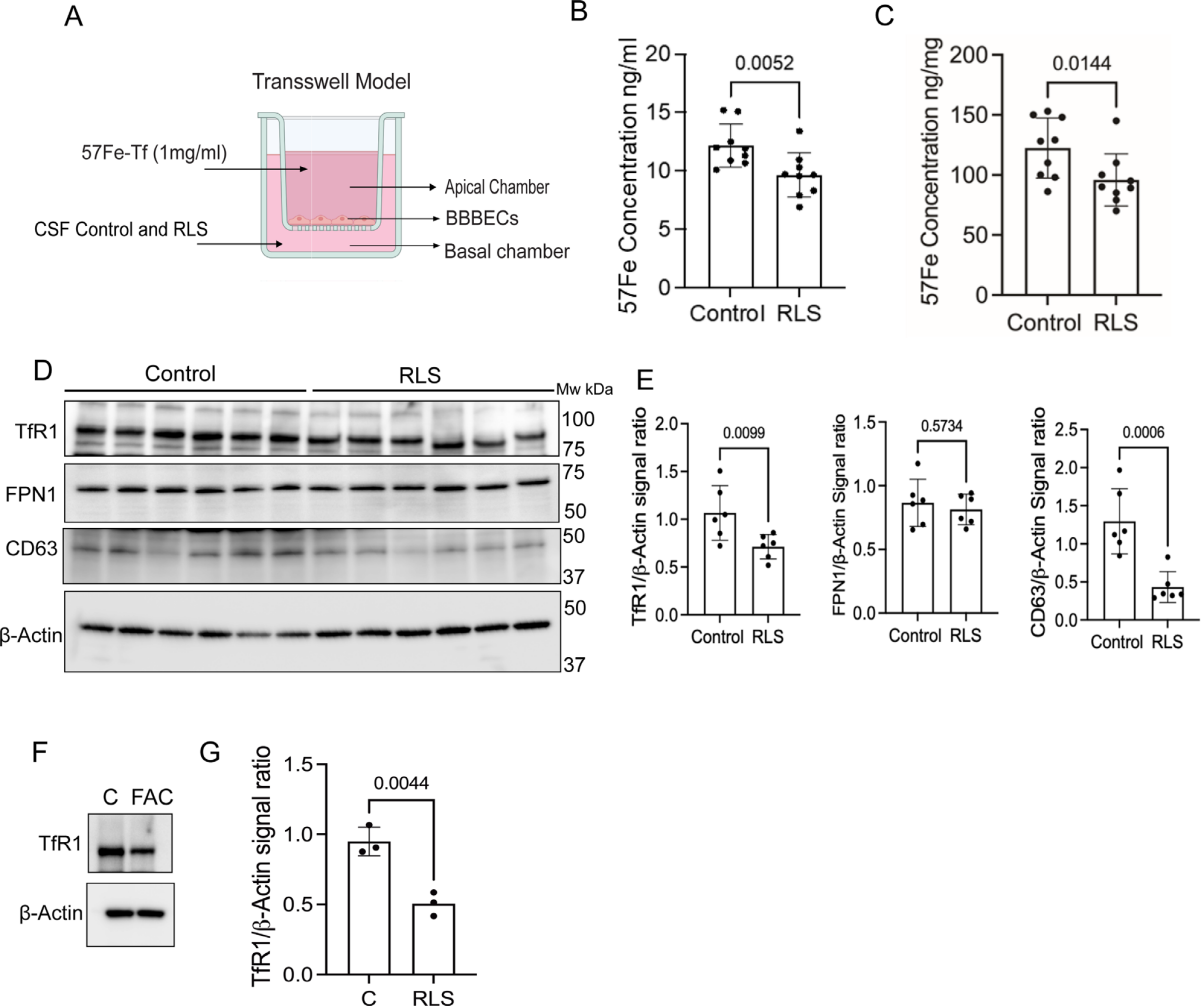
Restless legs syndrome (RLS) cerebrospinal fluid (CSF) reduces iron uptake and transferrin receptor 1 (TfR1) expression in the endothelial cells (ECs): ECs grown on the 0.4μm pore size filters were incubated with 25% of RLS and control CSF to the basal side and 1mg of ^57^Fe‐Tf was added to the apical chamber. After 24 hours, basal media and ECs lysate were collected. The ^57^Fe concentration was measured in media and cell lyase by inductively coupled plasma mass spectrometry (ICP‐MS). For iron treatment, the ECs were treated with FAC 100μM to the basal side and incubated for 24 hours and following incubation, the TfR1 expression was measured by immunoblot. (A), illustrates the experimental model of bichamber. (B) compares the basal medium ^57^Fe concentration in RLS and CSF‐treated ECs. (C), illustrates the comparison difference between the Tf‐^57^Fe uptake in RLS and controls CSF treated ECs. Data shown are the mean ± standard deviation (SD) of 3 independent experiments of n = 9 from control and RLS, unpaired *t* test (D). Representative cellular immunoblot analysis of iron‐responsive proteins transferrin receptor 1 (TfR1), ferroportin1 (FPN1), CD63, and β‐actin is present as the loading control. (E) Histograms for TfR1, FPN1, and CD63/β‐actin expressions and the data are shown as the mean ± SD of 3 independent replicates and analyzed by unpaired *t* test. Each band represents the pooled samples of n = 2 of control and RLS. (F, G) Immunoblot and bar graph represents the TfR1 expression in the ECs following basal side FAC treatment. The data are shown as the mean ± SD of 3 independent replicates and analyzed by unpaired *t* test. [Color figure can be viewed at www.annalsofneurology.org]

Next, we studied a model of the transport of iron to the brain using ECs exposed to RLS and control CSF. For this study, we pooled 2 samples based on their CSF total iron concentrations. We chose 2 samples from the lower group (below the average‐controls 98.7 ± 25, 110.47 ± 5.4; RLS 59.94 ± 9.81, 61.69 ± 2.52), 2 that fell at the mean value (control 131.10 ± 0.02, 127.32 ± 8.5; RLS 102.29 ± 1.37, 90.58 ± 27), and 2 from the higher (above the average of iron concentrations, control 156.41 ± 15.1, 174.01 ± 37.4; RLS 115.97 ± 1.62, 118.49 ± 1.9) iron concentration of the mean values of iron in control and RLS CSF samples. Next, we added the RLS and control CSF to the basal side of ECs, and Tf‐^57^Fe (1mg/mL) was added to the apical side of the ECs (Fig 2). After 24 hours of incubation, we measured the ^57^Fe levels in the basal media and ECs by ICP‐MS. The results demonstrate that transport of ^57^Fe to the basal media decreased significantly in RLS CSF‐exposed ECs than the control CSF. The iron transport depends on their uptake into the ECs cells. Next, we measured the uptake of ^57^Fe, in RLS‐CSF exposed ECs, and ^57^Fe concentration was lower compared to the control. Following the studies of Tf‐^57^Fe uptake and release, we determined the transporter expression in RLS and control CSF‐treated ECs. The TfR1, involved in the uptake of the Tf‐^57^Fe, was significantly decreased in RLS CSF‐treated cells compared to the controls, but there was no difference in the expression of FPN1, the free iron exporter. Last, we measured the expression of CD63, which mediates ferritin and transferrin‐bound iron release from ECs via exosomes.^16^ The RLS CSF exposure was associated with reduced expression of CD63 protein compared to the controls. Under conditions of lower iron levels in the brain, iron uptake is typically increased through upregulation of TfR1. However, in our study, despite reduced iron levels in the CSF of RLS patients, TfR1 expression was decreased. To demonstrate addition of iron would decrease the TfR1 expression, we exposed the basal side of the chamber to 100μM ferric ammonium citrate for 24 hours. Following incubation, TfR1 expression was measured, and basal iron exposure significantly reduced TfR1 expression compared with the control.

### RLS CSF Alters the Ferritin and IRPs Expression in the BBBECs

**FIGURE 3:**
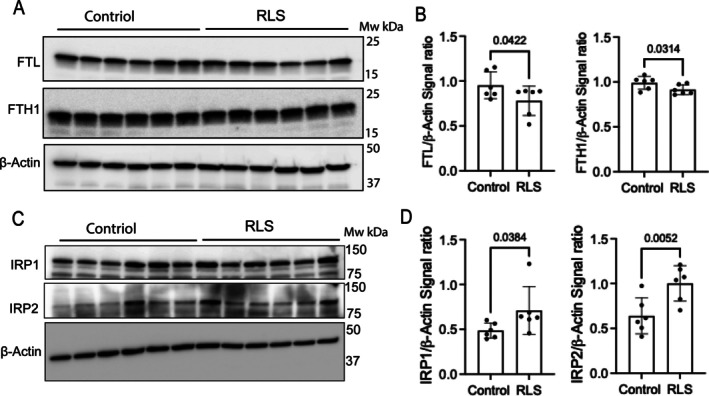
Restless legs syndrome (RLS) cerebrospinal fluid (CSF) alters the ferritin and iron‐regulatory proteins (IRPs) expression in the blood–brain barrier endothelial cells (BBBECs): endothelial cells (ECs) grown on the 0.4μm pore size filters were incubated with 25% of RLS and control CSF to the basal side. After 24 hours of incubation, cells were lysed in radioimmunoprecipitation assay buffer and measured the protein expression by immunoblotting. (A), illustrates immunoblot analysis of the iron storage protein expressions FTH1, FTL, and β‐actin is present as the loading control. (B) Histograms for FTH1 and FTL/β‐actin expressions (C) Represents immunoblots of the iron regulatory proteins IRP1 and 2, and β‐actin is present as the loading control, (D), histograms for IRP1 and 2/β‐actin expressions and the data are shown as the mean ± standard deviation of 3 independent replicates and analyzed by unpaired *t* test. Each band represents the pooled samples of n = 2 of control and RLS.

The expression of TfR1 depends on the cellular iron concentration.^28^, ^29^ Ferritin is an iron‐storage protein and a marker for iron levels.^30^ Therefore, we measured the level of expression of H and L ferritin in the ECs in both RLS and control CSF‐treated cells. The RLS CSF unexpectedly reduced the expression of both H and L subunits compared to the controls (Fig 3A,B). TfR1 and ferritin expression are regulated by IRPs.^19^ Therefore, we examined the expressions of IRP1 and IRP2. RLS CSF treatment significantly increased the expression of IRP1 and 2 compared to the controls (see Fig 3C,D).

### RLS CSF Reduces the IRP Binding Activity

**FIGURE 4:**
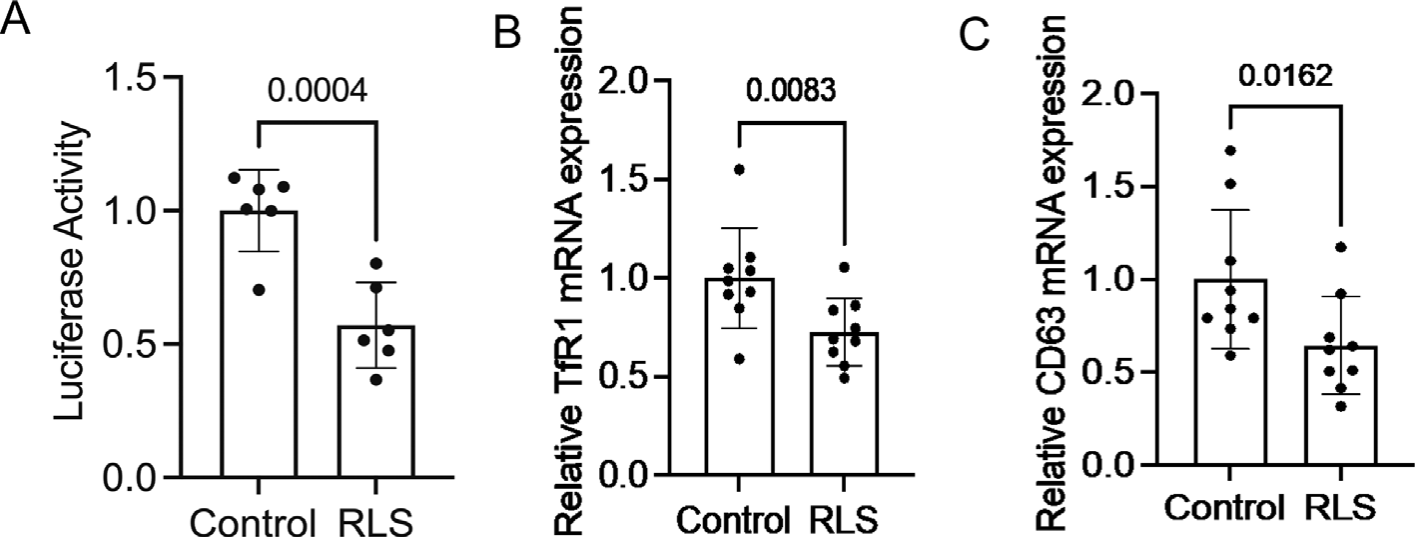
Restless legs syndrome (RLS) cerebrospinal fluid (CSF) reduces the iron‐regulatory protein (IRP) binding activity: endothelial cells (ECs) grown on the 0.4μm pore size filters were exposed with 25% of RLS and control CSF to the basal side. For determining the response of IRP binding activity in response to CSF treatment, we co‐transfected the luciferase and Renilla vectors with RLS and control CSF samples in HEK‐293 cells. (A) Illustrates the luciferase activity in HEK‐293 cells. (B, C) Represents mRNA expressions of transferrin receptor 1 (TfR1) and CD63 and β‐actin is present as the loading control and the data are shown as the mean ± standard deviation of 3 independent replicates n = 9 from RLS and control and analyzed by unpaired *t* test. For luciferase activity, samples of n = 12 of control and RLS. Each dot represents the 2 pooled samples.

The increase in IRPs associated with a decrease in TfR1 expression indicated there was a misregulation in this system. Therefore, we determined the IRPs binding activity because increased IRP expression and reduced ferritin levels are indications of iron deficiency in the ECs. During iron deficiency, TfR1 expression, however, should have increased, but its expression was decreased. The regulation of transferrin receptors is at the post‐transcriptional level by IRPs.^31^ To examine IRP binding to TfR1 mRNA IRE sequences, we cloned the 3′ IRE elements into the pMIR‐REPORT luciferase vector. To assess the stability of the 3′ IRE sequences, we conducted a luciferase assay in HEK‐293 cells. Cells were transfected with the luciferase vectors containing the 3′ IRE sequences and concurrently exposed to RLS or control CSF for 24 hours. Following incubation, luciferase activity was measured. CSF from RLS‐exposed samples resulted in reduced luciferase activity compared with cells treated with control CSF (Fig 4). The decrease in luciferase activity would be expected to result in decreased TfR1 mRNA. Therefore, we measured the TfR1 mRNA expression in RLS and control CSF‐treated ECs. The TfR1 mRNA levels were significantly decreased in RLS‐treated ECs. We further studied whether IRP's discordance with specific TfR1 mRNA IRE sequences or other iron‐responsive proteins. To confirm this, we measured another iron‐responsive protein CD63, which has a 5′ IRE sequence,^32^ RLS CSF treatment significantly reduced mRNA expression compared to the control treatment.

### RLS CSF EVs Associated miR‐124‐3p Have Functional Binding Activity to the TfR1 3′ IRE

**FIGURE 5:**
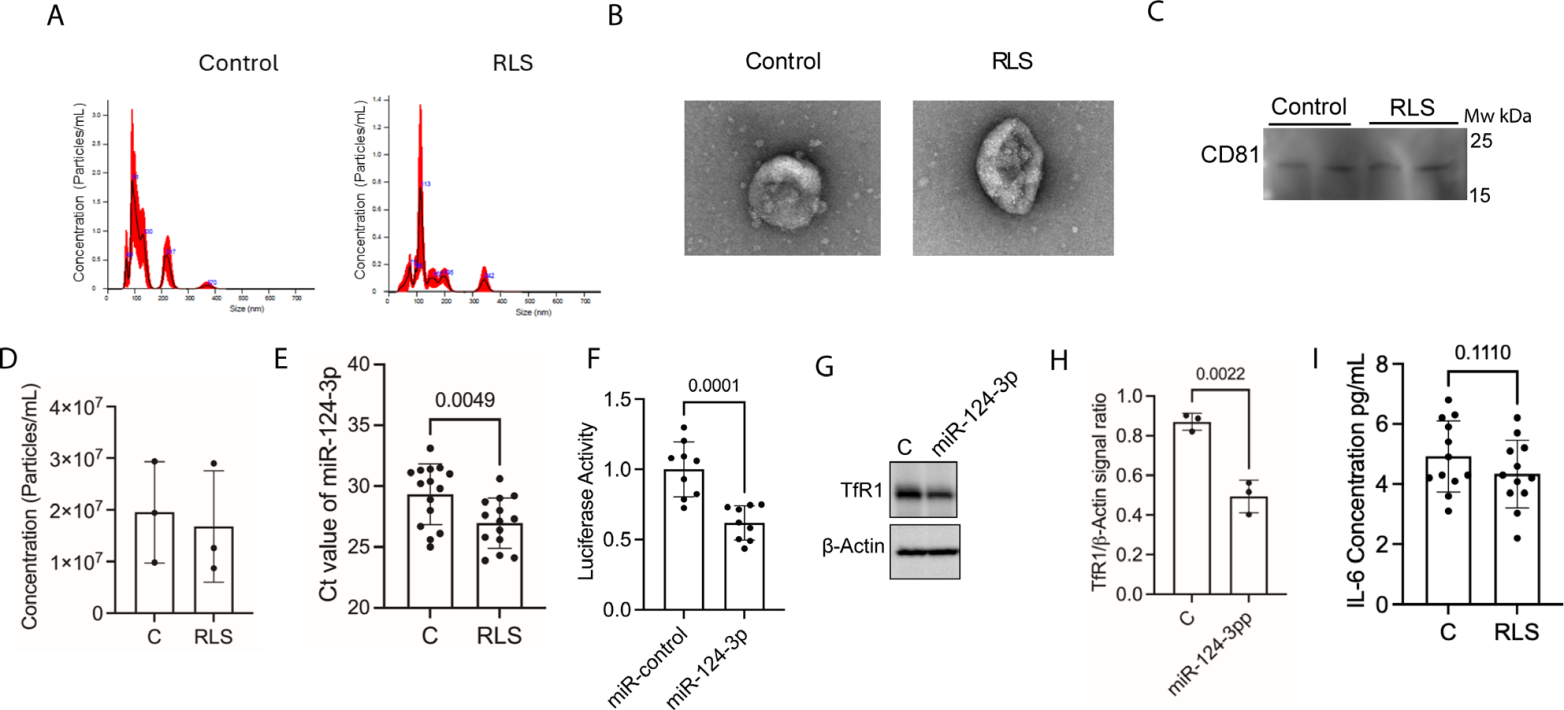
Restless legs syndrome (RLS) cerebrospinal fluid (CSF) extracellular vesicles (EVs) associated miR‐124‐3p have functional binding activity to the transferrin receptor 1 (TfR1) 3′ IRE. EVs were isolated from the control, and RLS CSF measured their associated miR‐124‐3p expression levels. For luciferase experiments, we co‐transfected 20nM of microRNA (miRNA) mimic‐miR124‐3p‐hsa‐mir‐124‐3p, miRIDIAN microRNA mimic negative control, and 1μg pMIR‐REPORTER luciferase containing ~700bp of TfR1 3′ IRE sequence in HEK‐293 cells, and for TfR1 protein expression, we treated only with miRNA mimic and negative. At 48 hours after transfection, luciferase activities were analyzed using the dual luciferase assay. Each miRNA transfection was done in triplets and independently repeated at least 3 times, resulting in at least 9 luciferase reporter assays for miRNA. (A) Representative image of nanoparticle tracking analysis of EVs sizes derived from the control and RLS CSF. (B) Transmission electron microscopy images of EVs shape from control and RLS. Negative staining of specimens was performed with uranyl acetate; the scale bar is 200nM. (C) Immunoblot of CD81, which is a membrane marker of EVs. (D) Bar graph illustrates the EVs concentration in control and RLS CSF. (E) Bar graphs represent the quantitative polymerase chain reaction results of miR‐124‐3p expression, which were expressed in Ct values. n = 15 control and n = 14 from RLS. (F) Bar graph illustrates the luciferase activity; the luciferase activity was normalized to the Renilla luciferase activity. G, H illustrates the TfR1 protein expression in HEK‐293 cells. β‐actin is present as the loading control, and the data are shown as the mean ± standard deviation (SD) of 3 independent replicates. (I) Interleukin ‐6 (IL‐6) concentration in control and RLS CSF. n = 12 from control and RLS. Data are shown as the mean ± SD and analyzed by an unpaired *t* test. [Color figure can be viewed at www.annalsofneurology.org]

Previous studies have shown that TfR1 mRNA expression is also regulated by miRNAs.^21^, ^22^, ^23^ To identify which miRNAs might interact with the TfR1 3′ untranslated region (3′ UTR), we used the miRNA target prediction algorithm, TargetScan.^33^, ^34^ Our analysis indicated that 8 miRNAs are interacting with 3′ IRE sequences. TargetScan predicts miRNA–mRNA interactions by identifying conserved sequences in 3′ UTRs. We further narrowed our candidates by miRNA expression in the brain and found that miR‐124‐3p was highly expressed in brain tissue. Studies of CSF have shown that miRNAs are predominantly transported between the brain cells via EVs,^35^ as free miRNAs are susceptible to degradation by RNases present in CSF. To determine whether the predicted miRNAs were present in EVs, we isolated EVs from RLS and control CSF samples and confirmed that isolated particles are EVs by their size, morphology, and EV‐specific membrane protein marker (Fig 5). CD81 is abundantly expressed in the EVs membrane and used as a marker for EVs. We also quantified EV numbers in the RLS and control groups and found no significant differences. After confirming the EVs isolation, we isolated their associated miRNA content and synthesized cDNA according to the manufacturer's protocol. We then measured miR‐124‐3p expression by qPCR by TaqMan probes. The Ct values for miR‐124‐3p ranged from 23 to 32, which falls within an acceptable range for qPCR detection. Notably, Ct values were significantly lower in RLS CSF compared with controls, indicating higher miR‐124‐3p expression in the RLS samples. Next, we performed a luciferase assay to determine whether miR‐124‐3p interacts with the TfR1 3′ IRE sequences. For this interaction, we inserted approximately 700bp of 3′ IRE sequences into the luciferase gene in the pMIR‐REPORTER vector and transfected it into the HEK‐293 cells with has‐miR‐124‐3p (miRNA mimic miR‐124‐3p) and miRIDIAN a control (negative miRNA), and measured the luciferase expression. Our results showed that mimic miR‐124‐3p significantly reduced luciferase activity compared to the control, supporting its functional interaction with the 3′ IRE region. Next, we measured the endogenous TfR1 protein expression in HEK‐293 cells with the treatment of miR‐124‐2p or control. We found that TfR1 protein expression significantly decreased in miR‐124‐3p‐treated cells than the control. Previous studies have shown that miR‐124‐3p acts as an anti‐inflammatory molecule.^36^ To examine whether this pattern was reflected in our samples, we measured the proinflammatory cytokine interleukin‐6 (IL‐6) in RLS and control CSF. Although IL‐6 levels were not significantly different between the groups.

## Discussion

The iron in the blood must undergo active transport across the BBB to enter the brain.^31^ The TfR1 on the brain's microvasculature plays a crucial role in regulating iron at the interface between the blood and CSF. Transferrin receptors are highly concentrated in the ECs that form the BBB.^32^ The transferrin–transferrin receptor complex is widely regarded as a key mechanism for transporting iron across the BBB. The current study shows that exposure to RLS CSF reduces iron uptake and releases in ECs compared to the control group, as well as decreases TfR1 expression in these cells. These findings align with our study of brain microvasculature isolated from human postmortem brains of RLS patients and controls, which also demonstrated lower TfR1 expression in RLS brains.^33^

The ability of the CSF from the RLS patients to impact the iron protein profile in the ECs is a novel finding and is in agreement with our previous study has shown that people with RLS have lower iron, ferritin, and higher transferrin levels in CSF than controls,^5^ which is a profile of iron deficiency. Here, we determined the amount of free and protein‐bound iron and found that both were decreased in RLS CSF. The decrease in free iron would be consistent with the iron deficiency induced by the RLS CSF in our cell culture model of ECs. Recently, we demonstrated iron–protein complexes released by ECs may be the predominant mechanism for iron release by the BBB.^12^, ^16^ This mechanism of iron release is also decreased in RLS CSF. Moreover, the decrease in protein‐bound iron is also concordant with the iron deficiency induced by the RLS CSF and corresponds with the decrease in CD63 expression. CD63 is an EVs marker involved in ferritin–iron release from ECs that is independent of ferroportin. Therefore, both iron release mechanisms into the brain are compromised in RLS.

In addition to compromised iron release, the data strongly suggest impaired iron uptake in ECs in RLS. This concept of impaired uptake is consistent with the decreased expression of TfR1 on the ECs after treatment with RLS CSF, but not control CSF. Exposure to RLS CSF induces an iron‐deficient state in the ECs, which should be met with an increase in TfR1, but our postmortem study^37^, ^38^ and the cell culture data in this study have found a decrease in TfR1 expression. Therefore, we proposed that RLS CSF contains signals that cause dysregulation in TfR1 expression. It is well established that IRPs play a major role in controlling the cellular iron homeostasis by post‐transcriptionally regulating the iron metabolic proteins.^39^ IRPs increase the TfR1 mRNA stability by binding to the IREs in 3′‐UTR, resulting with elevated TfR1 protein levels and increased iron uptake.^39^ In the present study, EC exposure to RLS CSF results in an apparent iron deficiency as indicated by lower H and L ferritin and higher IRP1 and 2 expressions, but, in contrast, there is reduced ECs TfR1 protein and mRNA expression. The increase in IRP levels suggested a possible discordance of post‐transcriptional mechanisms mediated by IRPs. Moreover, the discordance could be specific to TfR1 because the ferritin and CD63 levels in the cell decreased as expected in an iron‐deficient setting. Therefore, we studied the impact of RLS and control CSF on IRP1 and 2 activities. RLS CSF significantly increased IRP1 and 2 expressions in cell cytoplasm, but decreased luciferase activity in the HEK‐293 model. This finding suggests that RLS CSF is directly disrupting the IRP binding to the TfR1 3′ IRE sequences. The result of this disruption, as our data predicts, is a decrease in TfR1 mRNA and subsequent protein expression. The decrease in luciferase activity in the RLS CSF‐exposed HEK‐293 cells reveals a potential discordance between IRPs and 3′ IRE sequences of TfR1 mRNA. The genome‐wide association studies (GWAS) conducted in RLS patients to identify defects in genes involved in iron metabolism did not reveal any mutations in the TfR1 gene.^40^, ^41^ However, the GWAS method has the limitation of not accounting for post‐transcriptional changes in gene expression^42^ as we report here.

It is well known that miRNAs act as post‐transcriptional regulators of gene expression by binding to the 3′‐UTR, leading to translational repression or mRNA degradation.^43^, ^44^ miRNAs are a prominent class of small non‐coding RNAs, whose expressions can be altered during brain disease conditions and released into the extracellular space via EVs.^45^, ^46^ miR‐124‐3p is one of the most abundantly expressed miRNAs in neural cells and is also expressed in microglia.^36^, ^47^, ^48^ In our study, we observed that miR‐124‐3p levels were elevated in EVs from individuals with RLS compared with control CSF. We also found that miR‐124‐3p binds to 3′ IRE sequences and reduces luciferase activity, supporting its functional regulatory role in decreasing TfR mRNA. Therefore, we conclude that elevated miR‐124‐3p in RLS CSF triggers a decrease in TfR mRNA in ECs that result in brain iron deficiency. Further studies are needed to identify the conditions that increase miR‐124‐3p levels in the CSF of individuals with RLS. It is noted that miR‐124‐3p can be activated during inflammation, functioning as an anti‐inflammatory molecule and suppress microglial activation. Therefore, hypothesis to put forth for future testing is that RLS results from some form of inflammatory response (inappropriately robust or chronic low‐grade). We examined IL‐6 and tumor necrosis factor‐α levels in the CSF, but only IL‐6 proinflammatory cytokine levels were detected in the CSF by enzyme‐linked immunosorbent assay, and it was not different between control and RLS. We previously reported that cystatin C is elevated in the CSF of early‐onset RLS patients.^49^ This protein can be elevated in response to inflammation, suggesting further study into inflammatory profiles in RLS is warranted. Overall, these data strongly suggest that in RLS patients there is discordance of IRP/IRE interaction of the TfR1 mRNA in the ECs, which leads to reduction of the TfR1 mRNA stability, ultimately resulting in iron deficiency in the cell and decreased iron release into the brain.

In conclusion, our present results in the cell culture model are consistent with our previous findings from human postmortem tissue of lower TfR1 expression in brain microvasculature and neuromelanin cells.^37^, ^38^ These results are also concordant with the iron deficiency profiles in CSF.^5^ The current findings identify the possible mechanism of instability of TfR1 mRNA as the cause of the brain iron deficiency and provide a comprehensive understanding of the decreased brain iron levels in RLS. Our study also illustrates how a peripheral iron deficiency does not cause RLS, but the RLS brain has lower iron levels by decreasing the TfR1 expression in ECs, although peripheral iron deficiency could certainly contribute to the iron deficiency in the brain microvasculature. Moreover, we have identified future mechanistic targets to investigate miR124‐3p source and conditions that increase the release of signal molecules that lead to the discordance between the IRPs and the TfR1 mRNA.

## Author Contributions

K.P. and J.R.C. contributed to the conception and design of the study. K.P., A.P.S., L.M.T., and D.B.R. contributed to the data acquisition and formal analysis. K.P. and J.R.C. drafted a significant portion of the manuscript or figures.

## Potential Conflicts of Interest

L.M.T. is a member of the Board of Directors of the American Academy of Sleep Medicine (AASM) and AASM Foundation; J.R.C. is a member of the Scientific Advisory board of RLS, but had no involvement with any funding decisions related to the RLS Foundation award cycle in which K.P. was awarded (co‐sponsored by the AASM Foundation). Her role on this manuscript was as a co‐investigator on NIH R01NS113912.

## Data Availability

The datasets generated during the current study are available from the corresponding author on reasonable request.
